# Optimal Probe Length Varies for Targets with High Sequence Variation: Implications for Probe Library Design for Resequencing Highly Variable Genes

**DOI:** 10.1371/journal.pone.0002500

**Published:** 2008-06-18

**Authors:** Niall J. Haslam, Nava E. Whiteford, Gerald Weber, Adam Prügel-Bennett, Jonathan W. Essex, Cameron Neylon

**Affiliations:** 1 School of Chemistry, University of Southampton, Southhampton, United Kingdom; 2 Departamento de Fisica, Universidade Federal de Ouro Preto, Instituto de Ciências Exatas e Biológicas, Ouro Preto, Brasil; 3 School of Engineering and Computer Science, University of Southampton, Southhampton, United Kingdom; 4 STFC Rutherford Appleton Laboratory, Harwell Science and Innovation Campus, Didcot, Oxfordshire, United Kingdom; University of Liverpool, United Kingdom

## Abstract

**Background:**

Sequencing by hybridisation is an effective method for obtaining large amounts of DNA sequence information at low cost. The efficiency of SBH depends on the design of the probe library to provide the maximum information for minimum cost. Long probes provide a higher probability of non-repeated sequences but lead to an increase in the number of probes required whereas short probes may not provide unique sequence information due to repeated sequences. We have investigated the effect of probe length, use of reference sequences, and thermal filtering on the design of probe libraries for several highly variable target DNA sequences.

**Results:**

We designed overlapping probe libraries for a range of highly variable drug target genes based on known sequence information and develop a formal terminology to describe probe library design. We find that for some targets these libraries can provide good coverage of a previously unseen target whereas for others the coverage is less than 30%. The optimal probe length varies from as short at 12 nt to as large as 19 nt and depends on the sequence, its variability, and the stringency of thermal filtering. It cannot be determined from inspection of an example gene sequence.

**Conclusions:**

Optimal probe length and the optimal number of reference sequences used to design a probe library are highly target specific for highly variable sequencing targets. The optimum design cannot be determined simply by inspection of input sequences or of alignments but only by detailed analysis of the each specific target. For highly variable sequences, shorter probes can in some cases provide better information than longer probes. Probe library design would benefit from a general purpose tool for analysing these issues. The formal terminology developed here and the analysis approaches it is used to describe will contribute to the development of such tools.

## Introduction

There is currently an intense effort to improve the throughput as well as reducing the cost of DNA sequencing [1], [2]. It is hoped that the reduced costs from these new methods will make routine the sequencing of known genes for variants [3]. Conventional sequencing technologies are prohibitively expensive for large population based studies [3]. Many of the proposed approaches use sequencing by hybridisation (SBH) [4], [5], a technology based on the complementary nature of DNA to indirectly read a sequence. This technique employs a library of selected short oligonucleotide probes of known sequence composition with an identifying physical tag, usually the physical position on a 2-dimensional array. These probes are then brought into contact with an unknown target sequence. The successful hybridisation of the probes is then detected by a signal such as luminescence. The actual sequence composition of the hybridized probe is then retrieved by correlating the signal with the probe's tag. These techniques are all aimed at reducing the cost of DNA sequence analysis.

One application where cheap analysis has the potential to make a significant impact is in the sequence analysis of genes for drug targets in clinical samples [6], [7]. For example, the main drug targets in HIV, reverse transcriptase and protease, undergo mutation and selection for drug resistant variants in patients undergoing combination drug therapy [8]. It would be valuable to be able to determine the sequence variation in these genes within the viral population during drug therapy, and particularly when drug resistance becomes evident, so as to select the appropriate drug combination for the patient to move to. In these cases, the appropriate genes would be amplified, using PCR, from a blood sample, labelled and interrogated via SBH. For this to be viable the probe library used for SBH would need to be ‘generic’; capable of analysis of essentially all likely sample variation. This would keep the costs of production down but raises the question of whether a useful generic library can be built.

A standard approach is to use a tiling array which covers the entire gene and has all four possible variants at the central position of each probe [9]. This approach generally uses probes of length 15 to 30 nucleotides long. Such a probe library can essentially detect differences from the single template or ‘wildtype’ sequence used to generate the initial tiling array. However, for highly variable drug targets, where multiple sites of variation may be found within a single probe, for example adjacent SNPs, there is the potential for such ‘double mutations’ to lead to misleading results, both false negatives and in some cases potentially false positives [10], [11]. These approaches have therefore been very effective at identifying previously unknown sequence variation in DNA sequences that are not highly variable i.e. those where it is relatively unlikely that two mutations will occur in one probe. The approach to dealing with such double mutations is to identify sequence regions where none of the tiling probes have hybridised and to re-design the probe library or carry out targeted probe hybridisations to identify the local sequence. This approach is not viable for the situation we have outlined above where double mutations are extremely likely and the design, synthesis, and analysis of further probes will be costly when applied to a wide range of patients. Overlapping libraries provide an improvement over traditional array based resequencing in that they offer higher resolution sequence information [12]. In regions of the genes where more variability is observed more probes are generated to cover as much of the variation as possible.

An alternative approach is to attempt to capture all possible variation. The logical extreme of this is ‘universal’ libraries where the probe library contains all possible sequences of a given length, initially these used very short probe length, circa 5 nt, but more recent implementations have extended this to 10 nt [11]. These libraries require huge numbers (approaching 4^N^) of probes for relatively short probe length. For practically accessible library sizes, probe lengths cannot be easily extended beyond 10 nt [13]. For target sequences of any significant length this will not provide sufficient selectivity to uniquely identify specific sequences [11]. Here we show that even for relatively short sequences such as that of the HIV protease gene, sequences within the gene of up to 14 nt occur more than once making universal libraries inappropriate.

A third approach is to use known sequence variation to design a library. Even in genes that are known to be highly variable, such as drug targets in the HIV and influenza genome, the variation is often highly constrained. Specific variants occur at specific positions, generally with only two of the possible four bases observed at these positions, for example if the wildtype is C then only G mutants will be observed but not A or T. It is therefore possible to consider building a library that contains all possible sequence variants that can be formed by combinations of previously observed variants. The library would be constructed by comparing a set of previously determined reference sequences. A consensus sequence would be built making a note of all the observed variants at each position. Probes would then be designed by tiling along the consensus sequence with probes designed to hybridise to all possible combinations of variants that had been previously observed. Such a library would correctly identify more sequence variants than a traditional tiling library with variation at the single position where multiple mutations occur frequently within single probes and those mutations reflect a limited set of possible variants. However, the library would be limited to the detection of mutations that have been previously observed, i.e. any specific sequence variant not seen in any of the reference sequences will not be detected. Additionally the generation of all possible combinations may generate libraries with huge sizes. The effectiveness and practicality of using such libraries would therefore depend on a number of factors, the identity of specific variations that occur, their distribution along the gene sequence, the length of the gene, and the quantity and quality of reference sequences that are available for library design.

Here we investigate the ability of probe libraries generated by recombination of reference sequences to re-sequence highly variable genes, with a focus on viral drug targets. We show that such an approach is applicable to some targets, but that it will be unsuccessful for others. The potential effectiveness of these libraries is highly dependent on target identity, variability, composition and probe length, thermal filtering and the number of reference sequences used. A critical factor in the effectiveness of the libraries for each target is the probe length chosen. Probe length has two important consequences. Firstly, it determines in a simple way how many probes uniquely identify a specific sequence region within a target gene. Secondly, it has a very strong effect on the number of probes required to build the library. In some of the cases investigated here libraries based on reference sequences would outperform traditional tiling libraries. This combined with the ability to reuse these libraries for multiple analyses makes this an approach for library design with significant potential. However, for several of the targets analysed this approach will not work, making a detailed analysis of a specific target gene, such as that reported here, important before making a decision on the design approach. We define a number of variables that are important in determining the feasibility of sequencing a putative target sequence by sequencing by hybridization. In particular, we show some cases where sequencing by hybridization using reference sequences will not be feasible due to the amount of variability in the target sequence. In those cases where it is feasible we analyse the optimal probe length for a number of scenarios and show that this also varies wildly from target to target. Thus when applying similar re-sequencing approaches to highly variable target genes it is necessary to examine their viability and optimal design on a case by case basis. The analysis reported here provides a basis on which to consider and optimize the design of such probe libraries.

## Results

As case studies for this work we selected sequences from human immunodeficiency virus (HIV), hepatitis C (HCV) and influenza, all of which are highly variable organisms [14]. The selected genes from these organisms, summarised in Table 1, encode for important proteins, some of which are drug targets [7], and the variability of the gene has an important effect on the effectiveness of drug treatments. Many have SNPs that have been shown to be markers for resistance to drugs. Therefore, it is of practical importance and of general interest to reliably resequence these genes.

**Table 1:** Characteristics of the reference sequences used in this work.

Genome-subtype, gene	*N*_Ref_	L (nt)	*W*	*R*	*u*
HIV-A, reverse transcriptase (rt)	30	1320	286	0.122	2.177
HIV-A, protease (pr)	40	297	92	0.208	2.534
HCV-1a, E1	24	576	225	0.235	2.302
HCV-1a, core	22	573	89	0.101	2.458
HCV-1a, ns4a	25	162	103	0.526	3.145
Influenza-H3N2, np5	42	1521	101	0.041	2.342

Shown are the total number of reference sequences *N*_Ref_ used in this work, complete length *L*, number of variable positions W defined in eqn. 8, relative variability order 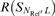 calculated from eqn. (6) and the geometric mean of the variations calculated from eqn. 9. Sequences were downloaded from the Los Alamos National Laboratory Sequence Databases [21].

In Methods we describe and develop a formal approach to describing and analyzing probe libraries and their relationship to target sequences which enables us to define a number of useful quantities in precise terms. For this work, we designed overlapping resequencing libraries *L_l_* by sliding a window of length *l* over a consensus sequence that covers each base in a gene several times. Subsequently, we retain only those probes that are thermodynamically equivalent using a method that we recently developed [15] (See Methods for a detailed description). The generation of an isothermic library should reduce the error rate of the sequence determination [16]. Once the libraries are built they are compared to a set of fragments *T_l_* of a selected target sequence that was not used as reference (See Methods). Therefore, the number of target fragments that are identified *t_f_* provides a measure of how effective the resequencing library is at detecting mutations. In particular, it allows us to study the effect of the number of reference sequences, *N_ref_*, used, as well as thermodynamic filtering and the dependency on probe length *l*.

To quantify the variability of a gene, we define a variability *V*(*S*) which is calculated for a set of *N* sequences *S_N,L_*, each of length *L* [17] (see Methods). In essence, this variability is the product of the number of different nucleotides that are observed at any given sequence position and represents the maximum number of different combinations one might expect to sequence based on the observed variation. Since this variability can be a very large number we also define the relative variability order,
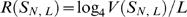
(1)


For instance, in this work the target gene *ns4A* is the most variable gene with a relative variability order of *R* = 0.526 and *np5* is the least variable with *R* = 0.041 (See Table 1). As a result of the high degree of variability in the sequences examined here (see Table 1), it is worth noting that the average mutation rate of once every six positions would result in probes of length 25 containing around 4 variable positions. This would result in an unacceptably high number of mutable positions leading to an explosion in the size of the library. More significantly if the library was restricted to only contain probes that varied at the central position then the rate of false positives and negatives would increase. Therefore, when resequencing this type of target then the library design must include probes with multiple variable positions.

### Repeated probes

**Figure 1:**
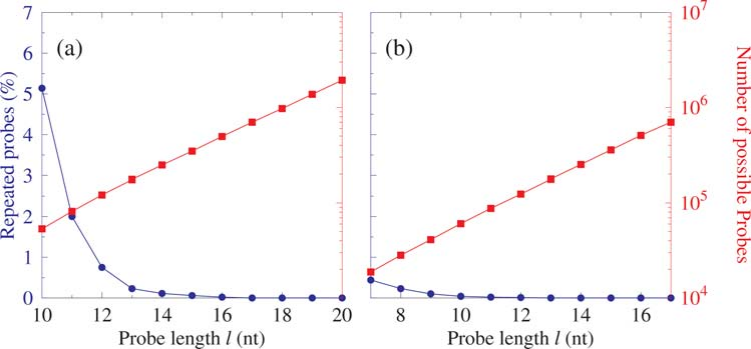
Percentage of probes that occur more than once in the raw library *L*. Fraction of repeated probes in the library 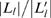 (blue bullets, left-hand scale) and total number of probes |*L_l_*| (red boxes, right-hand scale) as a function of probe length *l* in a) HIV reverse transcriptase and b) HCV *E1*. *L*′ is the reduced set of unique sequences as explained in Methods (See Equation 3). Lines are a guide to the eye, and it should be noted that the right hand scale is logarithmic.

The first problem which needs to be addressed for resequencing is that of sequence repetitions. We have previously shown that for a wide range of sequences the degree of repetition is strongly dependent on the probe length [18]. Initially, we create a library *L_l_*, for probes of length *l*, using the procedure outlined in the Methods section. As such this library may contain repeated probes which result from the fact that there may be multiple occurrences of specific sequences of length *l* present in the sequences that were used to build the library. Therefore, such a library introduces an intrinsic uncertainty when used for resequencing as it will not be possible to unambiguously assign the matched probes to a specific position within the target sequence. In Figure 1 we show some examples of the relationship between the amount of repeated probes in the raw library *L_l_* and probe length *l*. Usually, it is sufficient to select a large enough probe length *l* to remove any repeated probes. As the probe length increases, so does the number of probes, |*L_l_*|, in the library (Figure 1); as more sites of variation are covered by a single probe, potentially this could be a combinatorial expansion [17]. Therefore, it is possible to fine tune the length of the probes to avoid repetitions and to keep the size of the library to a minimum. The gene reverse transcriptase in HIV-A and *E1* in HCV both have a large relative variability as shown in Table 1. However, for *E1* combinations of mutations do not cause repetitions in the probe library after *l* = 12, whereas for HIV reverse transcriptase (RT) the probe length required to overcome repetitions is *l* = 16. As a result *E1* can be analysed with a much smaller probe library, than HIV RT, by using a shorter probe length, as shown in Figure 1.

This shows that optimisation of probe length is an important consideration in the design of resequencing libraries. The length of the probes used can have a significant impact on the type and quality of information retrieved in a sequencing by hybridisation experiment. The propensity of mutations in a target sequence to form non-unique reads and therefore the probe length required to overcome this is target specific. Errors that result from ambiguous reads are currently dealt with in the information processing after the experiment. Investigating these issues during the process of probe design could aid the information processing, as well as identifying potential pitfalls in experimental design.

### Number of Reference Sequences

**Figure 2:**
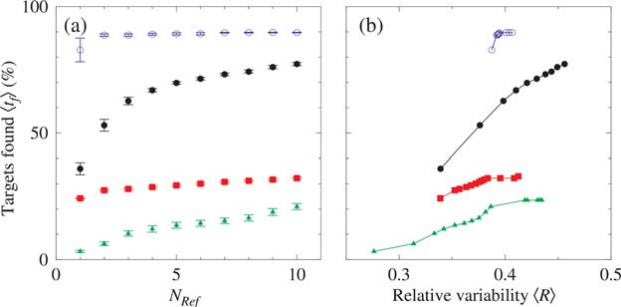
Relationship between variability and the number of targets found. Average fraction of targets 〈*t_f_*〉 found as a function of a) the number of reference sequences *N_Ref_* used and b) average relative variability of the library 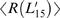 calculated for probe length *l* of 15 nt. Selected target sequences are for genes in HCV: *E1* (bullets), *core* (boxes), and *ns4A* (triangles) and Influenza *np5* (circles), NCBI∶AY746942, NCBI∶AY365214, NCBI∶D84264 and NCBI∶CY000172, respectively. The error bars in part a) represent the standard error in the mean and are the same for b). Lines connecting the symbols are guides to the eye.

While it is important to take into account a very large number of reference sequences for determining the optimal probe length, it is usually more practical to use a reduced set of reference sequences for designing the actual library. A reduced set of reference sequences results in a much smaller library which may still be effective for resequencing. This section will evaluate how many reference sequences are required to achieve adequate coverage (all sequences used are available in the supplementary material, see Text S1). To evaluate its effectiveness we compare the probes of each library to a selected reference sequence (the target sequence) that was not used in the design of the library. Specifically, we divide the target into overlapping fragments (*N_T_*) of the same lengths as the probes and determine how many of these match exactly to a probe in the library. Because repetitions have been avoided, as described previously, each matching probe relates unambiguously to a single position in the target sequence (unless previously unseen mutations generate a sequence that is repeated elsewhere in the probe library [11]). In Figure 2a) we show the average number of targets found 〈*t_f_*〉 for several genes as a function of the number of reference sequences *N_Ref_* used to build the library. The averages and standard errors are calculated over all possible libraries which can be designed with *N_Ref_* reference sequences out of the total available (see also Table 1); i.e. The process is repeated *N_Ref_* times using each reference sequence as a target and the remaining *N_Ref_ -1* sequences as the library. From Table 1, taking the number of variable positions and the length of the sequence it is possible to calculate on average how often a variable position will occur. For the sequences investigated here a variable position occurs on average every 5–6 bases. The least variable gene examined here is *np5* where a variable position occurs on average once every 15 bases. This means that for a probe, of length 25, designed to have only the central base probed that there will frequently be at least one additional mismatch per probe and possibly several. Such probes will require a more complex interpretation of the results as a consequence of the non-linear relationship between multiple mismatches in the same probe and hybridisation signal [10], [11]. Altering the probe length can overcome this problem by limiting the number of mismatches, due to the shorter length, or by interrogating more positions than the central base. Thus is it possible that *shorter* probes can be advantageous in analyzing highly variable sequences. This will be offset by the increasing probability that a probe sequences will be replicated elsewhere in the target sequence as the probe length drops, as shown in Figure 1.

The apparent saturation for *core* and *ns4a* (from Influenza) of targets found *t_f_* with increasing *N_Ref_* in Figure 2a) is deceptive. As shown in Figure 2b), the variability captured by the library is still increasing as *N_Ref_* is increased. However, this does not lead to an increase in *t_f_*. This is in contrast to *E1* and *np5* which tend toward some limiting value in both 〈*R*〉 and *t_f_*. Resequencing using sequencing by hybridisation is feasible for those targets where *t_f_* reaches a limiting value at a useful level with reasonable numbers of probes. For *np5*, 90% of targets are found with *N_Ref_* = 5. The average library size for *N_Ref_* = 5 is 4600. Therefore, in these cases using a small number of *N_Ref_* will minimise the size and therefore the cost of the library whilst maintaining the percentage of targets correctly identified by the library. By comparison, for *ns4a* with *N_Ref_* = 5 only 13% of targets are found. And even if the *N_Ref_* is increased to ten, doubling the average size of the library, only 20% of targets are found. This is clearly not high enough to make this approach viable for this target. Increasing *N_Ref_* does increase the library size; however, in the examples shown (*ns4a*, *core*), it does not lead to an adequate increase in the number of targets found. From Table 1, *ns4a* has the highest relative variability and this may explain the difficulty in capturing all possible variants. Therefore, a more conventional approach to resequencing will be required in this case.

Those targets in Figure 2b) that do not indicate any degree of saturation with regard to 〈*R*〉 or do indicate saturation but not at an acceptable level of 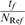 will be difficult to resequence using a sequencing by hybridisation library based on reference sequences. Resequencing these targets will be easier (and potentially cheaper) using other sequencing technologies based on direct detection methods. The variation found in these targets indicates that mutable positions are not shared from sequence to sequence and that therefore a much larger number of reference sequences and therefore larger probe libraries are required.

The examples in Figure 2 highlight the complex interplay between the variability of the target gene and that captured by the library as well as showing that the number of reference sequences required to build an effective resequencing library is target specific. This also gives an indication as to the optimal number of reference sequences to use to minimise the size of the library, and again that this number is target specific.

The difference in the number of reference sequences required to cover effectively a target shows that different types of variability may require different approaches. Sequencing by hybridisation may not be applicable to all types of resequencing applications. Our work provides a starting point for analysing the feasibility of short read resequencing to tackle some of these problems. This type of analysis is missing from current resequencing array design and it is clear from our results that there is a potential benefit to be gained from considering the number of reference sequences required. A reduction in library size, while retaining high coverage, can reduce costs. Our analysis provides an approach for measuring the likely effectiveness of sequencing by hybridisation and the optimal number of reference sequences for resequencing highly variable targets.

### Library Filtering

**Figure 3:**
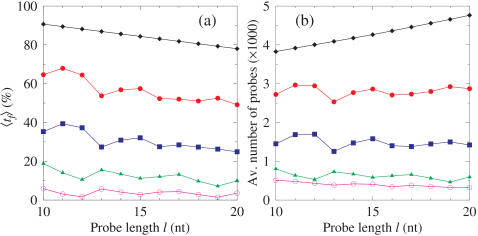
Effect of filtering on targets found. a) Number of fragments 〈*t_f_*〉 found the selected target sequence Influenza *np5* (NCBI∶CY000076) that are correctly identified as a function of probe length *l*. The average standard error is 0.23% and was consistent across all the data points. b) The average number of probes 〈|*L_l_*|〉 in the library as a function of probe length for each of the filtered libraries. The diamonds represent the library before applying the filtering. Remaining points represent the libraries where all sequences with *GC* content above 80% have been removed and thermodynamic filtering has been applied as described in Methods, with Δω = 1 (bullets), 0.5 (boxes), 0.2 (triangles) and 0 (circles). The average standard error was 9.5% of the library size and was consistent across all the data points. Lines are a guide to the eye.

For hybridisation experiments it is important to ensure that all probes hybridise selectively under the same conditions to ensure a minimum of false positives and negatives. Therefore, probes with melting temperatures that fall outside a specified temperature range need to be removed from the library since these probes are the most likely to give misleading results. The actual temperature range could be either a predefined experimental requirement or adjusted such that only the minimum number of probes are removed. For the temperature filtering we employ a new technique which we developed for evaluating the thermal equivalence of short DNA sequences [15]. In essence, this method calculates a simple index ω that can be related to the melting temperature of the probes (see Methods). This has advantages over traditional temperature calculations since this index does not depend on salt and species concentration [15]. The calculated equivalence parameter ω can be mapped to actual melting temperatures by the use of straightforward linear regressions, as described [15], once the remaining experimental conditions are established. Before applying the thermal filtering we remove all probes with a *GC* content higher that 80%, since such probes are usually difficult to synthesise and offer lower quality information [11], [19]. In Figure 3a) we show an example of the dependence of the number of targets found *t_f_* and the probe length, as well as the effect of thermal filtering, for Influenza *np5*. For unfiltered libraries, the number of targets found *t_f_* decreases slowly and monotonically with longer probes. However, for thermal filtering we notice oscillations in *t_f_* mainly due to the discrete nature of the equivalence parameter ω as well as due to an uneven distribution of this parameter along the gene. The number of targets found when only the probes with high GC content are removed is very similar to the number of targets found with no filtering and therefore is not drawn. Figure 3b) gives the size of the library before and after the filtering process.

**Figure 4:**
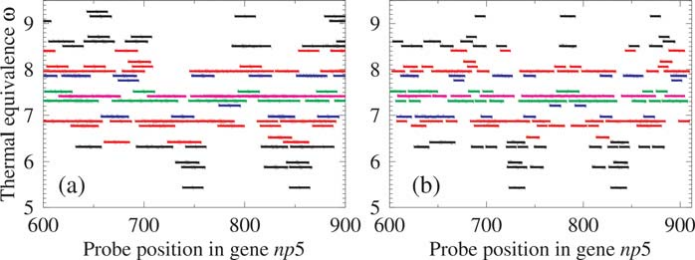
Equivalence parameters of probes in libraries and targets. a) Equivalence parameter ω of probes in the library (*L*_12_ and *N_Ref_* = 5) as a function of the sequence position *m* of the probe in the target sequence (*np5*). b) The equivalence parameter ω of probes that intersect the library and an unseen target *T*_12_(*NCBI*∶*CY* 000172) as a function of the sequence position *m* of the probe in the target sequence. The equivalence parameter ω is defined in eqn. 11 The beginning (*m*) and end (*m*+12) of the probe is shown by short up and down lines, respectively. The colour coding is: black for Δω>1, red for 0.5<Δω≤1, blue for 0.2<Δω≤0.5, green for 0<Δω≤0.2 and magenta for the most frequent equivalence parameter ω*_f_* or Δω = 0.

Figure 4a) shows how the equivalence parameter ω is distributed in a library along a section of the gene (*np5*) and which probes would be removed by the different thermal filtering criteria (For the sets of filtered probes see Text S2). This shows that even if only a small number of target fragments are found by the filtered library, if these targets are evenly distributed along the gene these should be sufficient for resequencing. A very strict thermal filtering causes the strongest depletion of probes from the library, however as long as the thermal equivalence parameter ω of these probes are not strongly clustered around certain positions in the gene it is still possible to obtain a reasonably uniform coverage. In Figure 4b) we show how the parameter, ω, for probes that find their target are distributed in a section of the gene Influenza H3N2 *np5*. These results illustrate the coverage that would be achieved in resequencing Influenza *np5* using different thermal filtering criteria. The figure highlights regions of the target susceptible to incorrect or unsuccessful probe hybridisation due to having a thermal equivalence parameter that varies too far from the library average.

**Figure 5:**
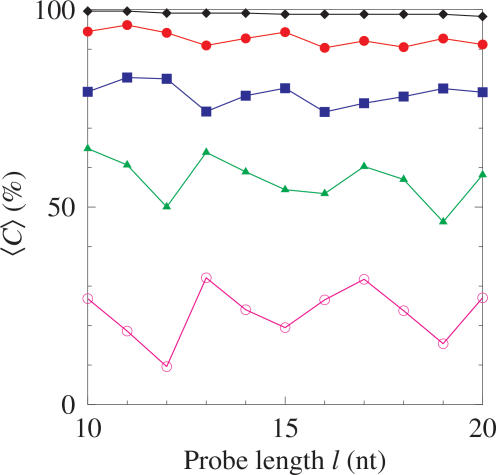
Percentage coverage after filtering. Average coverage by probes of gene positions 〈*C*〉 as a function of probe length *l* for Influenza H3N2 *np5*. The diamonds represent the unfiltered library and the remaining symbols represent the libraries filtered as described in Methods. Values for Δω are 1 (bullets), 0.5 (boxes), 0.2 (triangles) and 0 (circles). Lines are a guide to the eye. The average standard error is 0.5% and was consistent across all the data points.

We define a coverage *C* of positions in the gene covered by at least one probe which allows us to evaluate how well distributed the filtered probes of a given library are. Figure 5 shows this coverage *C* for the same gene Influenza H3N2 *np5* as function of probe length and several situations of thermal filtering. Without filtering almost all positions are covered even for longer probes, e.g. 99.3% for 20 nt. Similar to the number of targets found, the coverage *C* depends strongly on the selected probe length and with strong thermal filtering no simple rule can be deduced. Currently, error adjustment from differential hybridisation characteristics resulting in differential hybridisation signal intensities is carried out after the experiment. Our filtering method could inform the likely difference in intensity since the probes furthest away from the optimal hybridisation temperature could be identified beforehand and assumptions about their ability to hybridise could be built into the design *a priori*.

The filtering analysis demonstrates the effect on coverage and the number of targets found when altering the stringency of the filtering criteria. DNA hybridisation experiments are typically carried out using isothermic libraries. Our analysis shows the dependency of coverage on the width of the temperature band used to filter the library. With this information it is possible to make adjustments in the design for the required level of coverage to compensate for the greater deviation in temperature and the effect this may have on the reliability of some of the probes.

The results for each of the genes studied are summarised in Table 2. The difference in optimal probe length required to overcome repetitions and to maximise coverage, even between genes in the same organism highlights the need for a general purpose tool capable of resolving the conflicting requirements. To take the example of *np5* in Figure 5, the overall optimal probe length, with Δω = 0, is 13 giving coverage of 32%. This reflects the length required to overcome possible repetitions and maximises the coverage of the filtered library. Interestingly if Δω is increased to 0.5, then the overall optimal probe length would be 12, giving coverage of 82%; and finally if Δω was increased to 1 then the optimal probe length would be 15, giving coverage of 94%, as shown in Figure 5. This shows that there is no simple relationship between the optimal probe length and stringency of thermal filtering. Determining the optimal probe length with regard to filtering stringency is non-trivial and strongly target dependent.

**Table 2:** Optimal Probe lengths for genes studied.

Genome, target name	Minimal *N*_Ref_	Unique length	Optimal isothermal length	Coverage (%)
HIV-A, reverse transcriptase (rt)	5	16	16	48.1
HIV-A, protease (pr)	5	17	17	21.2
HCV-1a, E1	6	13	19	9.0
HCV-1a, core	21	17	18	13.8
HCV-1a, ns4a	24	14	14	8.6
Influenza-H3N2, np5	5	12	13	31.7

Shown are the optimal number of reference sequences *N*_Ref_, the length at which all possible subsequences are unique i.e. 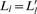, the optimal probe length upon filtering with Δω = 0 and the percentage coverage using these parameters.

## Discussion

We have examined a number of highly variable genes that are desirable targets for a cheap and effective re-sequencing technology. Sequencing by hybridization is an appealing approach for such targets but the variability of these genes precludes the use of traditional tiling libraries. In this work we have examined the potential of probe libraries based on information on known variation to solve this problem. In specific cases (e.g. Influenza *np5*, and HIV *rt*) such libraries can provide useful levels of sequence information (>90% coverage). However, in several cases the nature of the variation make this approach not viable (coverages less than 30% for *ns4a* and *core*). Where it is viable there is a complex interplay between the requirement to both reduce the number of probes in the library, and reduce the number of variable positions in each probe, which tends to lead to shorter probes, and the requirement to ensure that the majority of probes are unique within the target sequence, which tends to lead to longer probes.

Our central finding is that the optimal probe length varies significantly from target to target. Thus for any given highly variable target it is not straightforward to predict, by simple inspection of the sequence or of alignments, either whether such a probe design approach will work at all, nor what the optimal probe length is. In addition the optimal probe length is dependent on the stringency of thermal filtering applied in the library design process.

The relatively small number of targets examined here show clearly that probe library design for highly variable target genes will need to proceed on a case by case basis. A detailed consideration of the required sequence coverage, the degree of stringency in thermal filtering, and the resources available for probe library construction, will be required before any decisions can be taken on probe length, or indeed whether SBH is an appropriate approach to use.

The type of design analysed here can overcome, in specific cases, some of the shortcomings of traditional tiling probe libraries in which the central position of 25 nt probe is varied; specifically when resequencing DNA that varies at more than one position in each probe. When such variation occurs the central probe based designs will be unable to distinguish between the hybridisation intensity signal of single and double mismatches leading to false positives and false negatives. Only varying the central position of the probe will never be able to achieve the required coverage for the re-sequencing of highly variable genes because of the ambiguity of the results. Allowing more positions to vary, whilst significantly increasing the size of the library, is the only way for sequencing by hybridisation techniques to overcome this problem. To mitigate the effects of allowing more positions to vary the probe length must be decreased, from the more commonly used lengths of between 15 to 30 nt, to make the library size practical. Universal arrays represent a possible solution for re-sequencing of highly variable genes but increasing the probe length to above 10 nt increases the number of probes required above and beyond the capacity of a standard array.

The analysis presented here shows that adopting an intermediate length makes it possible to use re-sequencing arrays on some highly variable targets. The traditional tiling array approach is applicable work for the cases where the variability of the target sequence is not as high as those sequence examined here. However, if the application of sequencing by hybridization is to be extended into re-sequencing of variable viral drug targets then we suggest the probe length and variability should be considered as part of a feasibility test.

We present here an analysis that provides a means of investigating the feasibility of such an approach and the effect of probe length on in the design of an optimised library. A reduction in the size of a probe library will lead directly to cost reductions in the production of the library. In some cases identified here this reduction can be achieved without compromising the effectiveness of the library in interrogating a previously unseen target. The optimisation of probe length and number of reference sequences will need to be considered on a case by case basis. Using the analyses presented here it is possible to determine the sequences for which this will be feasible. As increasing quantities of sequence information are generated by SBH and microarray technologies it is important to consider both efficient experimental design and the potential pitfalls of the technology. Probe design tools based on the analysis described here have the potential to contribute to an improvement in the implementation of SBH experiments.

The software used to carry out the analysis is available at http://sgenomics.org/haslam/variability.tgz or alternatively from the authors on request.

## Methods

### Basic definitions

#### Sequences and sets

We write the *k*-th sequence of length *L* as
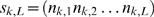
(2)where *n_k,j_* represents the nucleotides *A*,*C*,*G* and *T*. The multiset *S_N,L_* containing *N* of such sequences of length *L*, is then defined as
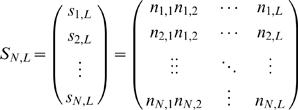
(3)


The superscript ^*^ stands for the complementarity; i.e., n^*^ is the complementary nucleotide of *n*, s^*^ the complementary sequence of *s* and S^*^ the multiset of complementary sequences of the sequences in *S*.

The set *S*′ is the reduced set of unique sequences in *S*, i.e., *S*′ contains no repeated sequences.

The cardinality of the multiset *S*, i.e. the number of elements that it contains, is indicated as |*S*|. In particular, if the multiset has no repeated elements then |*S*′| = |*S*|.

#### Variability

From eqns. (2) and (3), the multiset *S_N,L_* can be written as
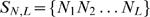
(4)where *N_i_* is the subset of nucleotides at position *i* of each sequence,
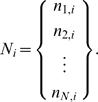
(5)We define variation 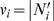 as the cardinality of the reduced set 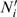, i.e. number of different nucleotides found in the subset *N_i_*. The total variability *V* of the multiset *S_N,L_* can then be calculated as
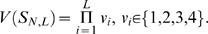
(6)


The limit values for the variability are *V*(*S_N,L_*) = 1 if all sequences are identical and *V*(*S_N,L_*) = 4*^L^* if all four nucleotides occur at all positions. This assumes independence between sites or linkage equilibrium. Therefore, since the variability may take very large numbers for large *L*, we introduce, for practical purposes the logarithm to base 4 of the relative variability order (*R*),
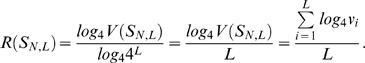
(7)Since the maximal value the variability *V* can take is 4*^L^*, the maximal for *R* will be 1.

To complete the characterisation or the variability of sequence sets we also define the number of variable positions
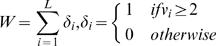
(8)which allows us to define the geometric mean of the variations which are larger than 2,
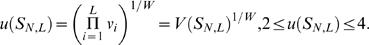
(9)


For example consider a set of *N* = 3 sequences of length *L* = 7,
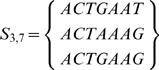
we only have two positions with more than one nucleotide, i.e. *W* = 2, which are
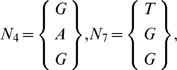
and their reduced sets would be
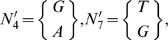
Therefore, considering the variations at all positions we would arrive at a variability of *V*(*S*_3,7_) = 4, relative variablity order *R*(*S*_3,7_) = 1/7 and geometric mean of the variation is *u* = 2.

The characteristics of the sequences used for building the libraries in this work are summarised in Table 1, along with the calculated parameters defined in this section.

#### Consensus sequence

Extending the procedure to calculate the variations of a set of sequences *S_N,L_*, the consensus sequence *s_cons._* can be obtained from
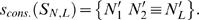
(10)


For the example set *S*_3,7_ of the previous section, the consensus sequence would be
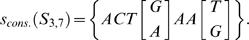



#### Library of probes

We can now define a set of probes *P_l,m_* of length *l* starting at nucleotide position *m*

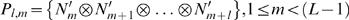
(11)From these probes we can form a new set of sequences, now of length *l*,
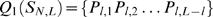
(12)


The library *L_l_*, for probes of length *l*, for a set *S_N,L_* is then formed out the set of sequences *Q_l_* and their complementary set as
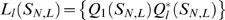
(13)Since the same probes may occur in different sets *P*, the reduced library *L*′ may be smaller than *L*. This reduction in size is due to the intrinsic repetition of the sequence as well as combinations of mutations resulting in non-unique probes and is discussed in Fig. 1.

Using the example set *S*_3,7_ of the previous sections, we could form a library *L* with probes of length *l* = 4 nt,
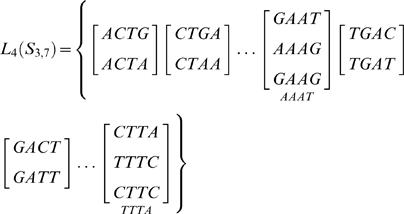



#### Target sequences

For each gene we chose one particular sequence at random to serve as the test target sequence *s_t,L_*. This target sequence is not included in any of the *N_Ref_* reference sequences used for building the libraries. We obtain a set *T* of subsequences of size *l* for this target sequence by using eqn. (13), i.e. *T_l_* = *L_l_*(*S_t,L_*) a ‘library’ formed of only one sequence. Since we are dealing only with one sequence, the number of test subsequences can be obtained immediately as |*T_l_*| = 2(*L*−*l*). The fraction of test subsequences found is the cardinality, i.e. the number of elements of the intersection of *T_l_* and the library 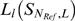


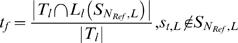
(14)


#### Averages

If we have *N* sequences at our disposal, we can build
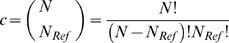
different libraries by combining *N_Ref_* sequences. In this work we take the averages by calculating a given quantity for all possible libraries and represent these averages by angle brackets. For instance the average of the relative variability order 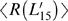 would have been calculated for all possible libraries 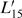 that can be built, or the average of the number of targets found 〈*t_f_*〉 would be all *t_f_* calculated for each of the *c* possible libraries that can be formed with *N_Ref_* reference sequences.

### Library filtering

#### Thermal equivalence

To evaluate if probes in a library will hybridise at the same temperature range we use the approximate form of the thermal equivalence parameter ω [15],
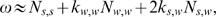
(15)where *N_a,b_* are the number of nearest-neighbours of type (*a*,*b*). Base-pair types are grouped according to the number of hydrogen bonds, i.e., with strong *s* and weak *w* representing *CG* and *AT* base-pairs respectively. The sum of the number of all nearest-neighbours is simply the length of the probe, i.e., 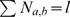. In this work we used *k_s_*_,*w*_ = 0.4795 and *k_w,w_* = 1.205 as described in [15]. Note that, within this model, the first and last base-pairs of the sequence are also counted as nearest-neighbours and as a result *N_s,w_* = *N_w,s_* [15]. The parameter ω provides a more generic way of evaluating the thermodynamic equivalence of DNA sequences since, unlike the melting temperature, it is essentially independent of salt and species concentration [15]. Effectively, this allows the selection of probes that have similar hybridisation temperatures under most experimental conditions. In this work, the thermodynamic filtering is carried out within an interval ±Δω around the most frequent equivalence parameter ω*_f_* in the library. For a sufficiently symmetric distribution the most frequent equivalence parameter ω*_f_* will usually be a value very close to the mean, i.e. ω*_f_*≈〈ω〉 in most cases.

While the thermal equivalence ω depends on the length of the probe *l*, the filtering interval Δω can be used independently of probe length. Consider *n_a_*_,*b*_ to be the changes to the number of nearest-neighbours *N_a_*_,*b*_, such that
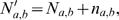
(16)and if the probe length is unchanged 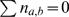. Therefore, it is straightforward to show that the difference between two probes of the same length with indexes ω and ω′ will be
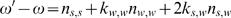
(17)where we used the property *n_s_*_,*w*_ = *n_w_*_,*s*_. Note that eqn. (17) does not depend on the probe length *l*. In other words, any pair of probes of the same length and which differ in configuration by the same set of changes {*n_a_*_,*b*_} will have the same difference in equivalence parameter ω′−ω independently of the probe length itself. This is an important result since it means that the same Δω can be used to filter probes of any length, e.g. using Δω = 0.5 is not more or less restrictive if the probe length is 10 or 20 nt. As a result, the thermal filtering for a given set of probes depends only on the statistical distribution of the values of ω.

Since probes with a high *GC* content have been shown to be difficult to synthesise and have a low discriminatory power (increasing the likelihood of cross-hybridisation), in this work we remove probes with *GC* content above 80% [19], [20].

## Supporting Information

Text S1 Target sequences used. The additional file Sequences.fa.tar contains the sequences used to carry out these experiments. Each file in the archive has the sequence data that we used to create the library for each target sequence. Each file in the archive corresponds to one target, for example, protease.fasta corresponds to the data we used to analyse HIV protease.
(0.01 MB TAR)

Text S2 Example probe library generated by this analysis. This additional file Probes.fa.tar contains an example of a probe library set, for the gene np5 at read length 17, generated in this analysis. The files are labeled in a format to indicate the stringency of filtering (GC content followed by Δω). These are also available from the author and on the 4g.soton.ac.uk website.
(0.17 MB TAR)
